# Topoisomerase I inhibitor, irinotecan, depletes regulatory T cells and up-regulates MHC class I and PD-L1 expression, resulting in a supra-additive antitumor effect when combined with anti-PD-L1 antibodies

**DOI:** 10.18632/oncotarget.25830

**Published:** 2018-07-31

**Authors:** Toshiki Iwai, Masamichi Sugimoto, Daiko Wakita, Keigo Yorozu, Mitsue Kurasawa, Kaname Yamamoto

**Affiliations:** ^1^ Product Research Department, Kamakura Research Laboratories, Chugai Pharmaceutical Co., Ltd., Kanagawa, Japan

**Keywords:** PD-L1, PD-1, irinotecan, Tregs, combination

## Abstract

Anti-PD-L1 antibodies inhibit interactions between PD-L1 and PD-1 and interactions between PD-L1 and B7-1, thereby reinvigorating anticancer immunity. Although there are numerous ongoing clinical studies evaluating combinations of standard chemotherapies and anti-PD-L1 antibodies, irinotecan has not yet been investigated in this context so there is little information about its compatibility with anti-PD-L1 antibodies. Here we investigated the efficacy of anti-PD-L1 antibody in combination with irinotecan and the role of irinotecan in the tumor–immunity cycle in an FM3A murine tumor model. Despite a transient decrease in lymphocytes in the peripheral blood after irinotecan treatment, the antitumor activity of anti-PD-L1 antibody plus irinotecan was significantly greater than each agent alone. Irinotecan in combination with anti-PD-L1 antibody enhanced proliferation of CD8^+^ cells in both tumors and lymph nodes, and the number of tumor-infiltrating CD8^+^ cells was higher than either irinotecan or anti-PD-L1 antibody monotherapy. Irinotecan was found to decrease the number of Tregs in lymph nodes and tumors, and specific depletion of Tregs by anti-folate receptor 4 antibodies was found to enhance the proliferation of CD8^+^ cells in this model. In addition, irinotecan augmented MHC class I expression on tumor cells and concurrently increased PD-L1 expression on tumor cells and tumor-infiltrating immune cells. These results indicate that irinotecan may enhance the effect of T cell activation caused by anti-PD-L1 treatment by reducing Tregs and augmenting MHC class I–mediated tumor antigen presentation, and concurrent upregulation of PD-L1 expression can be blocked by the anti-PD-L1 antibody. These interactions may contribute to the superior combination effect.

## INTRODUCTION

Programmed death ligand 1 (PD-L1) is expressed on tumor-infiltrating immune cells and on cancer cells, where it plays a major role in suppressing the host’s antitumor immune response [1]. Its receptors are programmed death 1 (PD-1) and B7-1 (also known as CD80), which are expressed on effector T cells. Interaction between PD-L1 and PD-1 or between PD-L1 and B7-1 delivers signals that inhibit the antitumor activity of T cells [2]. Anti-PD-L1 antibodies that inhibit the interaction between PD-L1 and PD-1 and between PD-L1 and B7-1 have been shown to reinvigorate host’s anticancer immunity. Anti-PD-L1 antibodies have been shown to provide clinical benefit and be of acceptable tolerability in patients with urothelial carcinoma and non–small cell lung cancer [3, 4]. However, anti-PD-L1 antibody monotherapy induces long-lasting disease control in only some patients, and there are rising hopes for combination therapies comprising anti-PD-L1 antibodies plus other therapeutic agents [1]. The limited activity of anti-PD-L1 antibody monotherapy might be explained by the presence of suppressive factors in the cancer–immunity cycle [5].

Studies on a broad range of tumor cells *in vitro* and in syngeneic mouse tumor models have shown that some chemotherapeutic agents inhibit these suppressive factors and/or activate the immune system response. Therefore, combination therapy with anti-PD-L1 antibodies plus chemotherapy is considered a potentially valuable approach [6]. However, a major disadvantage of chemotherapy is its lack of specificity: Any proliferating cell—not only tumor cells but also lymphocytes—will be susceptible to chemotherapy-induced cell death, and lymphopenia is one of the main reasons why chemotherapy and immunotherapy have been seen as mutually antagonistic treatment options [7]. Nevertheless, there are numerous clinical studies evaluating combinations of standard chemotherapeutic agents plus PD-L1/PD-1 inhibitors.

Irinotecan, a topoisomerase 1 inhibitor, is a chemotherapeutic agent widely used for the treatment of a variety of cancers, including small cell lung cancer, gastrointestinal cancer, and breast cancer [8–11]. However, the role of irinotecan in the tumor–immunity cycle has not yet been investigated and there are few clinical studies evaluating the combination of irinotecan with PD-L1/PD-1 inhibitors.

In this study, we investigated the efficacy of irinotecan in combination with an anti-PD-L1 monoclonal antibody (PD-L1 mAb) by using a syngeneic mouse tumor model, and we investigated the targets upon which irinotecan acts to activate antitumor immunity and which may contribute to the combination effect of irinotecan plus anti-PD-L1 therapy.

## RESULTS

### Combination therapy with irinotecan plus PD-L1 blockade improved tumor control compared with monotherapy

**Figure 1:**
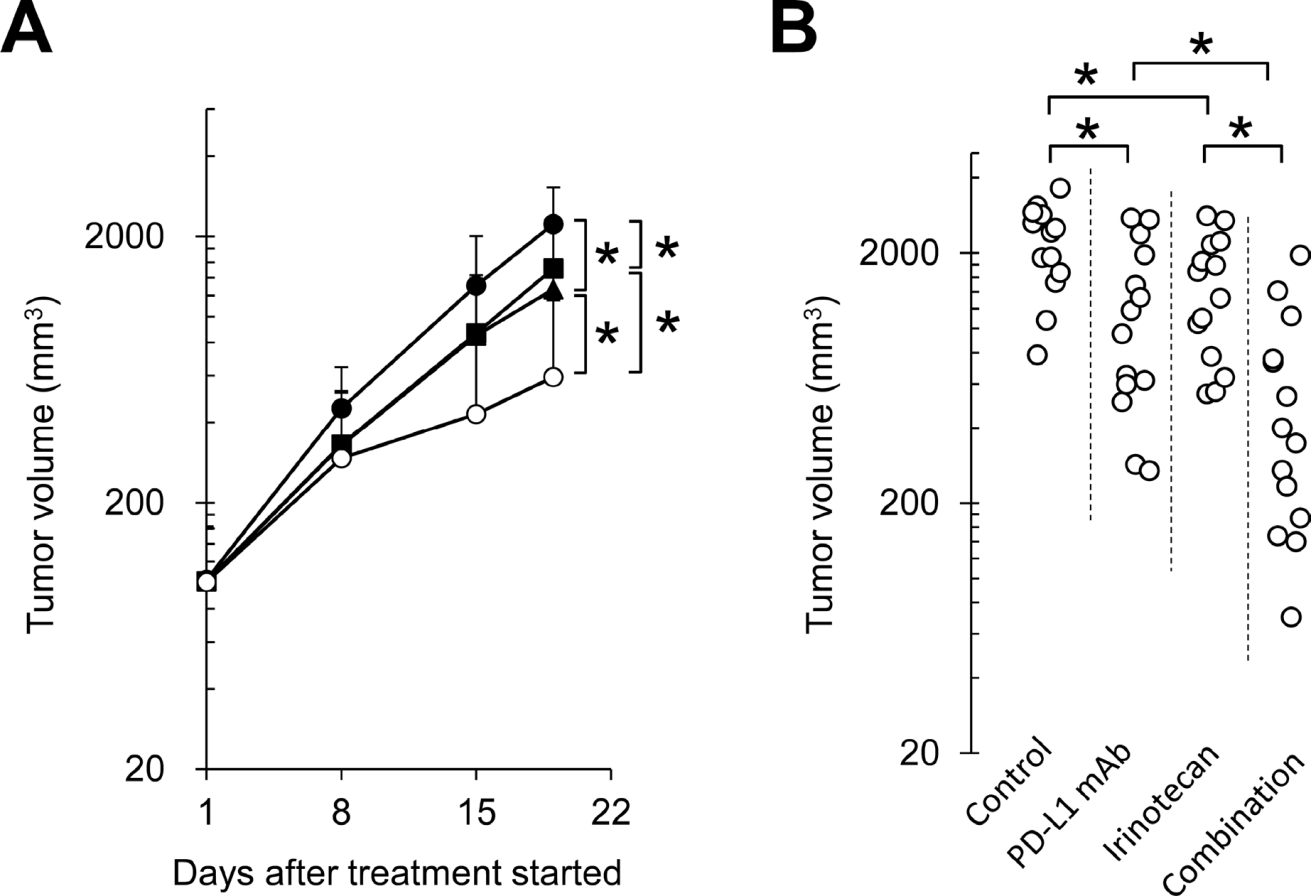
Combination therapy with anti-PD-L1 antibody and irinotecan inhibited tumor growth in the FM3A syngeneic tumor model (**A**) Tumor growth curve: Mice bearing FM3A tumors were randomly divided into groups. ●: control, ■: irinotecan 250 mg/kg, ▲: PD-L1 mAb 10 mg/kg, ○: combination. (**B**) Individual actual tumor volumes at the end of the study (Day 19). Data are shown as the mean + SD (*n* = 13–14/group). Statistical analysis used Wilcoxon rank sum test and the method of Holm.

To examine the combination effect of irinotecan plus PD-L1 mAb *in vivo*, FM3A tumor-bearing mice were administered irinotecan and PD-L1 mAb either as single agents or in combination. Irinotecan or PD-L1 mAb administered as a single agent each significantly inhibited tumor growth compared with the control group (Figure 1). Notably, the antitumor effect of irinotecan plus PD-L1 mAb was significantly greater than with either monotherapy (Figure 1). At the end of the study (Day 19), the mean growth in tumor volume in the PD-L1 mAb group was approximately 58% of the mean growth in tumor volume in the control group, 69% for the irinotecan group, and 27% for the combination therapy group. Therefore, the joint action of the combination of irinotecan with PD-L1 mAb was determined to be supra-additive (0.27 < 0.69 × 0.58) (Table 1).

**Table 1:** The joint action of the anti-PD-L1 antibody plus irinotecan combination in the FM3A syngeneic tumor model

	Control	PD-L1 mAb (*P*)	Irinotecan (I)	Combination
Mean tumor volume on Day 1 (mm^3^)	103	101	101	101
Mean tumor volume on Day19 (mm^3^)	2226	1265	1514	593
Relative effect (θ)	-	0.58	0.69	0.27
θ(P) × θ(I)	-	-	-	0.40

### Combination therapy with irinotecan and PD-L1 blockade increased number of intratumoral CD8^+^ T cells

**Figure 2:**
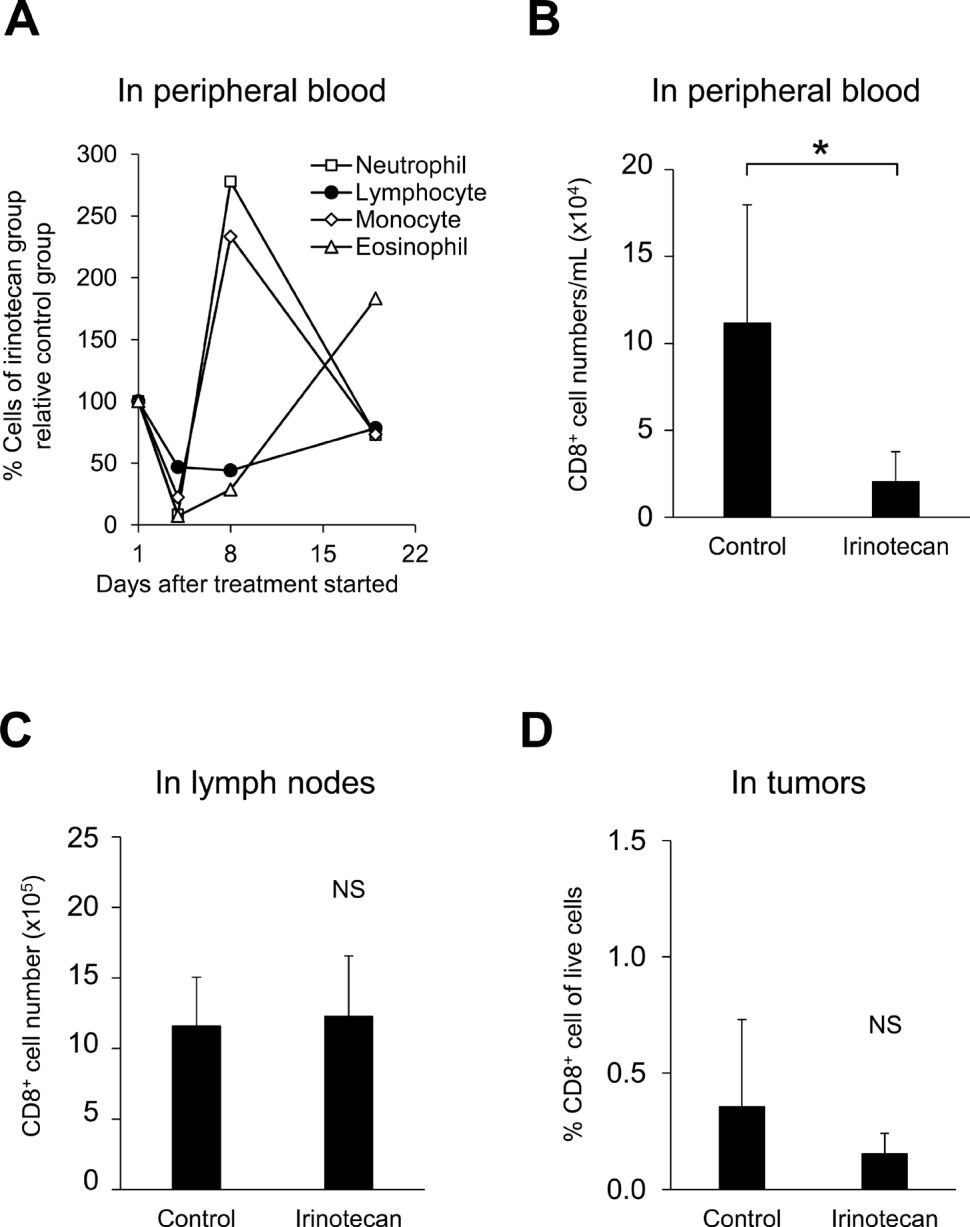
Irinotecan induced peripheral hematotoxicity but did not decrease the number of CD8^+^ T cells in tumors or lymph nodes (**A**) General hematologic analysis in the peripheral blood: hematologic indices were measured with an automated hematology analyzer. Irinotecan was administered on Day 1. Data are shown as the mean (*n* = 11–14/group). Analysis of CD8^+^ T cells on Day 8 in (**B**) peripheral blood (*n* = 6/group), (**C**) lymph nodes (*n* = 12/group), and in (**D**) tumors (*n* = 12/group). CD8^+^ T cells were determined by flow cytometric analysis. Data are shown as the mean + SD. ^*^p < 0.05, Wilcoxon test.

Clinical studies show that irinotecan induces leukopenia including lymphopenia [12]. In this FM3A murine breast tumor model study, neutrophils in the peripheral blood were decreased on Day 4 after administration of irinotecan alone but recovered rapidly with a transient increase on Day 8 (Figure 2A). On the other hand, lymphocytes in the peripheral blood also decreased on Day 4 and remained decreased over a long period (Figure 2A), thus we were concerned that the antitumor effect of PD-L1 mAb via T lymphocytes would be impaired. Therefore, we analyzed the number and the proliferation status of T cells in the peripheral blood, lymph nodes, and tumors on Day 8 after administrating irinotecan and found that irinotecan significantly reduced the number of CD8^+^ T cells (Figure 2B) and CD4^+^ T cells (data not shown) in the peripheral blood, however in both tumors and lymph nodes, the number of CD8^+^ T cells (Figure 2C, 2D) and CD4^+^ T cells (data not shown) was not decreased.

**Figure 3:**
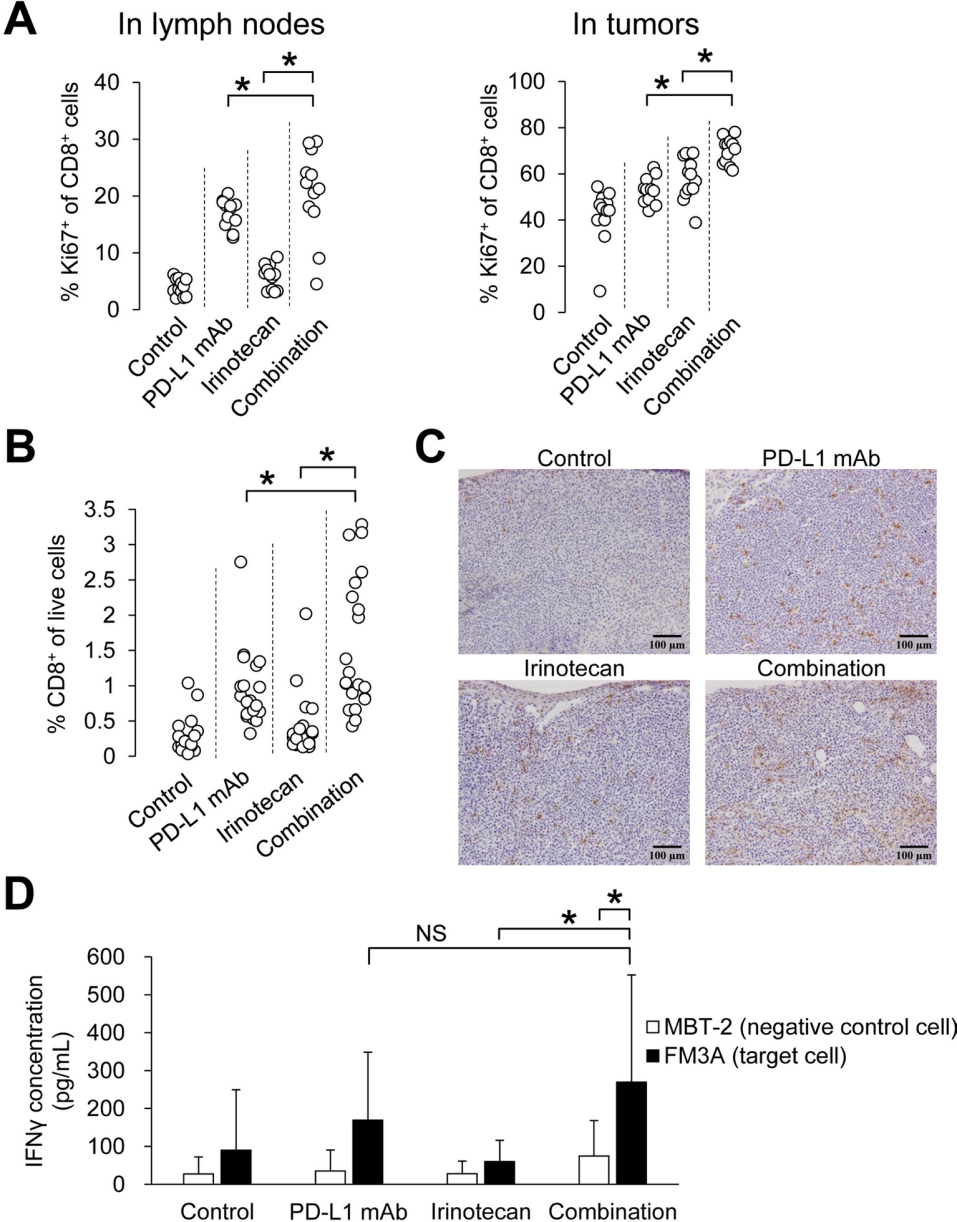
Combination of irinotecan plus PD-L1 mAb enhanced proliferation of CD8^+^ T cells and increased number of tumor-infiltrating CD8^+^ T cells without loss of PD-L1 blockade-induced tumor-specific lymphocyte response (**A**) Proliferation of CD8^+^ T cells in lymph nodes and tumors on Day 8 (*n* = 12/group). (**B**) Percentage of CD8^+^ T cells in tumor at the end point of the study (Day 19) (*n* = 19–21/group). CD8^+^ T cells were determined by flow cytometric analysis. (**C**) Infiltration of CD8^+^ T cells in tumors was determined by CD8α immunostaining in tumor tissue at the end point of the study (Day 19). (**D**) Secretion of IFNγ after specific stimulation of lymphocytes by co-culturing with tumor cells (*n* = 21/group). IFNγ was quantified by ELISA. Data are shown as the mean + SD. Statistical analysis used Wilcoxon rank sum test and the method of Holm.

Of note, the percentage of Ki67^+^CD8^+^ cells (proliferating CD8^+^ T cells) in the irinotecan plus PD-L1 mAb group significantly increased compared to that in each monotherapy group in both lymph nodes and tumors on Day 8 (Figure 3A), and the percentage of CD8^+^ T cells in tumors was significantly increased in the combination group compared with that in the PD-L1 mAb or irinotecan monotherapy groups at the end of the study (Day 19) (Figure 3B). These results were also confirmed immunohistochemically (Figure 3C).

Next we investigated the tumor-specific T cell responses during combination therapy. We prepared lymphocytes from tumor-draining lymph nodes on Day 19, and analyzed the release of IFNγ when the lymphocytes were stimulated by co-culturing them with either FM3A tumor cells or with MBT-2 cells (negative control). IFNγ in the combination group was significantly increased in the FM3A-stimulated group compared to that in the MBT-2-stimulated group, and among the FM3A-stimulated groups, IFNγ in the combination group was significantly increased compared with that in the irinotecan group (Figure 3D).

### Irinotecan reduced the number of Tregs and increased the proliferation of CD8^+^ T cells

Since irinotecan used in combination with PD-L1 mAb clearly increased the proliferation of CD8^+^ T cells in tumors and lymph nodes (Figure 3A), we next focused on the effect of irinotecan on myeloid derived suppressor cells (MDSCs) and regulatory T cells (Tregs), both of which are known to suppress CD8^+^ T cell proliferation [13, 14].

**Figure 4:**
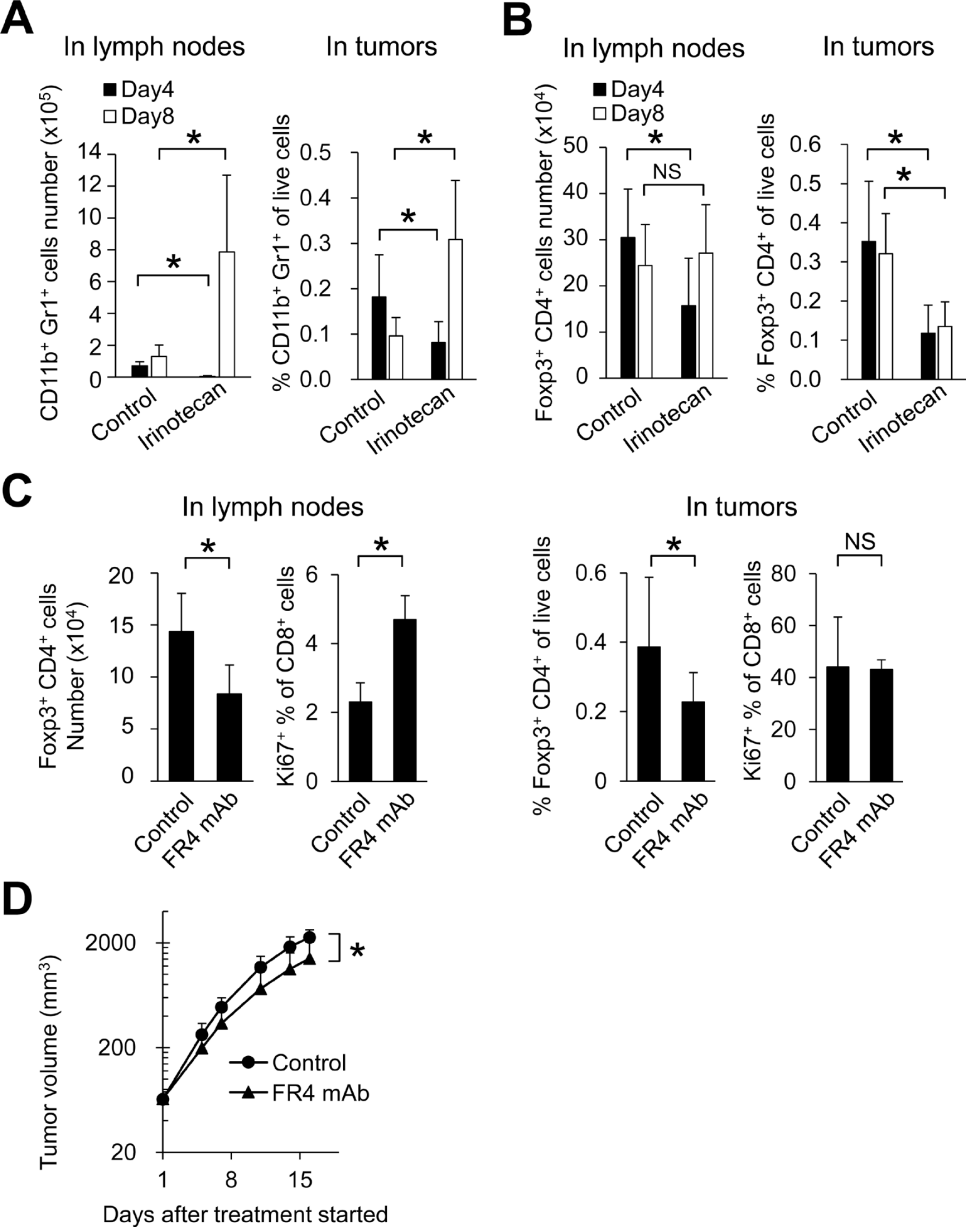
In lymph nodes, irinotecan increased CD8^+^ T cell proliferation through Tregs depletion Number of (**A**) MDSCs and (**B**) Tregs in lymph nodes and tumors on Day 4 and Day 8 after irinotecan treatment (*n* = 6–12/group). (**C**) Number of Tregs and proliferation of CD8^+^ T cells after FR4 mAb treatment in lymph nodes and tumors on Day 8 (*n* = 5–6/group). (**D**) Antitumor activity of FR4 mAb in the FM3A tumor model (*n* = 7/group). Data are shown as the mean + SD. ^*^p < 0.05, Wilcoxon test.

First, we checked whether irinotecan affected the number of MDSCs and Tregs in the early phase soon after treatment in the FM3A tumor models. Although the number of MDSCs (CD11b^+^Gr1^+^ cells) was significantly decreased on Day 4 after administration of irinotecan, by Day 8 the situation had reversed and MDSCs were significantly increased in both lymph nodes and tumors (Figure 4A). With Tregs (Foxp3^+^CD4^+^ cells) on the other hand, the number of Tregs in tumors and lymph nodes was significantly decreased on Day 4 after administration of irinotecan. In tumors, reduction of Foxp3^+^CD4^+^ cells by irinotecan administration was maintained until at least Day 8 (Figure 4B). These results indicate that although irinotecan initially depletes both Tregs and MDSCs, depletion of Tregs is maintained over a longer period.

Subsequently, to investigate the effect of Tregs depletion on the proliferation of CD8^+^ T cells and tumor growth in the FM3A model, we administered FR4 mAb, an antibody that causes Tregs depletion [15]. FR4 mAb decreased the number of Foxp3^+^CD4^+^ cells in both tumors and lymph nodes (Figure 4C). FR4 mAb significantly increased the percentage of Ki67^+^CD8^+^ cells in the lymph nodes not but in tumors (Figure 4C). Administration of FR4 mAb resulted in significant tumor growth inhibition (Figure 4D).

### Irinotecan increased MHC class I expression but also PD-L1 expression in tumors at the same time

**Figure 5:**
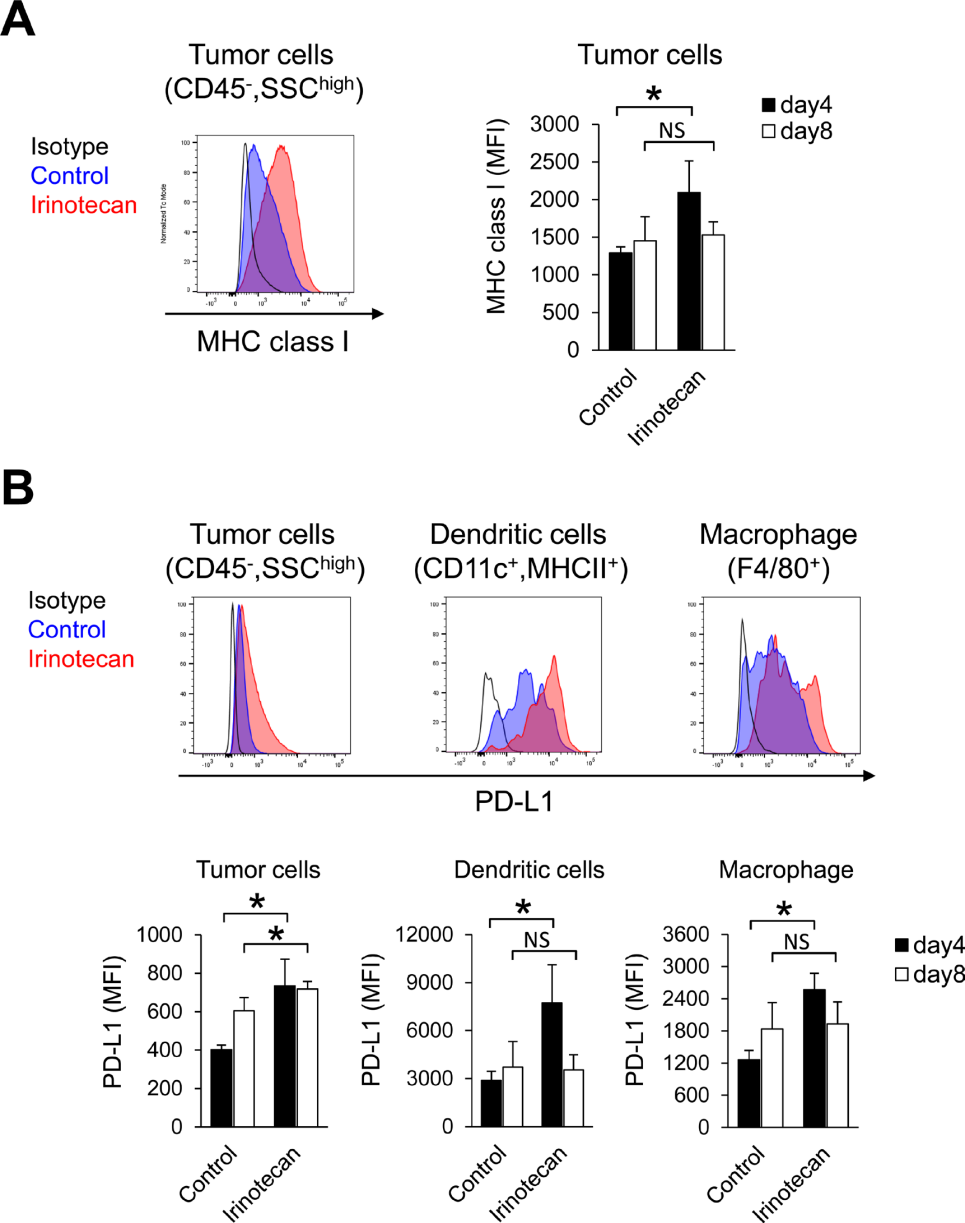
In tumors, irinotecan increased MHC class I expression but also PD-L1 expression at the same time (**A**) MHC class I expression on tumor cells on Days 4 and 8. (**B**) PD-L1 expression on tumor cells and on tumor-infiltrating dendritic cells and macrophages on Days 4 and 8. MHC class I and PD-L1 was determined by flow cytometric analysis. Data are shown as the mean + SD (*n* = 6/group). ^*^p < 0.05, Wilcoxon test.

Because Tregs depletion alone did not increase the proliferation of CD8^+^ T cells in tumors, we hypothesized that irinotecan may have the potential to activate other immune responses in addition to Tregs depletion in tumors. Therefore, to examine whether treating tumor cells with irinotecan upregulates other immune molecules implicated in CD8^+^ T cells activation, we analyzed MHC class I (H-2D^k^) and PD-L1 expression on tumors in the early phase after treatment. Expression of H-2D^k^ on untreated tumor cells (CD45^−^, SSC^high^) was low (Figure 5A). Irinotecan treatment resulted in upregulated H-2D^k^ expression on the tumor cells on Day 4 but not on Day 8 (Figure 5A). Irinotecan treatment resulted in upregulated PD-L1 expression on tumor cells and on tumor-infiltrating dendritic cells and macrophages on Day 4, but by Day 8 PD-L1 expression was upregulated only on tumor cells (Figure 5B).

## DISCUSSION

Immune checkpoint inhibitors like PD-1/PD-L1 mAbs have become standard therapies for patients with several tumor types; however, the treatment response only occur in a subset of patients [16]. Although there are numerous clinical studies evaluating the combination of PD-L1/PD-1 inhibitors with standard chemotherapeutic agents to extend their capacity, irinotecan has not yet been investigated in this context and there is little information about its compatibility with PD-L1/PD-1 inhibitors [17]. With irinotecan, similar to other chemotherapeutics, one of the dose-limiting toxicities is hematotoxicity, with leukopenia occurring in over 90% of the patients who receive irinotecan-containing regimens [18]. This raises concerns that the effect of PD-L1 mAb via T cell activation would also be impaired. In this murine tumor model we showed that irinotecan did indeed induce CD8^+^ T cells lymphopenia in the peripheral blood but not in lymph nodes or tumors (Figure 2B, 2C). Recently, it was reported that hypoxia-induced transcription factors contribute to the chemoresistance of tumors to irinotecan or etoposide [19, 20]. Hypoxia in the tumor microenvironment may cause resistance to irinotecan not only for tumor cells but for CD8^+^ T cells in tumors by promoting the anti-apoptotic and pro-survival mechanisms of CD8^+^ T cells.

Interestingly, irinotecan plus PD-L1 mAb increased intratumor CD8^+^ T cells possibly via accelerating CD8^+^ T cell proliferation in lymph nodes and/or tumors, and the combination of irinotecan plus PD-L1 mAb showed stronger anti-tumor activity than did PD-L1 mAb alone. It is widely known that Tregs inhibit growth and activation of CD8^+^ T cells [13]. Our study showed that irinotecan decreased Foxp3^+^CD4^+^ T cells in tumors and lymph nodes (Figure 4B). These results indicate that the depletion of Tregs by irinotecan may be one of the mechanisms underlying the increased proliferation of CD8^+^ T cells within tumors and lymph nodes. This is supported by the selective depletion of Tregs by FR4 mAb which reduced Tregs to similar level as irinotecan (Figure 4C). It is reported that Foxp3^+^ Tregs in lymph nodes are distributed diffusely in the T cell zone where CD8^+^ T cells accumulate [21]. Therefore, in the lymph nodes Tregs may effectively suppress effecter T cells. On the other hand, FR4 did not increase the percentage of Ki67^+^CD8^+^ T cells in tumors. Therefore, we hypothesized that irinotecan exerts effects in tumors other than decreasing Tregs. We showed that irinotecan transiently upregulated MHC class I on tumor cells (Figure 5A) in addition to decreasing Tregs in tumors (Figure 4B), it also upregulated PD-L1 expression on tumor cells and immune cells, such as macrophages and dendritic cells (Figure 5B). These results indicate that irinotecan may suppress the host’s antitumor immune response via up-regulation of PD-L1 on tumor cells, regardless of the increase in antigen-presentation from tumor cells via up-regulation of MHC class I. Therefore, blockade of PD-L1 by the combination treatment may allow the intrinsic immune system to exploit the irinotecan-enhanced antigen stimulation. Actually, it has been reported that several chemotherapeutic agents directly or indirectly increase PD-L1 and MHC class I, and show a synergistic effect when combined with anti-PD-L1 therapy [22–26]. The expression of PD-L1 and MHC class I on FM3A cells was increased by IFNγ but not by irinotecan *in vitro* (data not shown); therefore, irinotecan may upregulate PD-L1 and MHC class I expression indirectly by induction of host cell mediated-cytokines such as IFNs. It has been reported that another topoisomerase I inhibitor, topotecan, also upregulated the expression of MHC class I on tumor cells and topotecan-exposed tumor cells were more susceptible to T cell-mediated cytotoxicity [27]. Therefore the combination effect with anti-PD-L1 antibody might be generally applicable to topoisomerase I inhibitors.

In conclusion, we showed that combination therapy of PD-L1 mAb plus irinotecan exerted supra-additive anti-tumor activity in a preclinical tumor model despite the fact that irinotecan induced CD8^+^ T cell lymphopenia. A possible mechanism is that irinotecan enhances T cell activation caused by anti-PD-L1 therapy possibly by reducing the number of Tregs and by augmenting expression of MHC class I on tumor cells, and at the same time, the anti-PD-L1 antibodies can block the irinotecan-induced PD-L1 in tumors and lymph nodes. The present study may provide a rationale to conduct clinical studies of anti-PD-L1 antibodies in combination with topoisomerase I inhibitors, irinotecan, in small cell lung cancer, gastrointestinal cancer, and breast cancer.

## MATERIALS AND METHODS

### Cell lines and culture conditions

Cells of the murine mammary cancer cell line FM3A were obtained from the RIKEN Bio Resource Center (Tsukuba, Japan) and were maintained at 37° C in 5% CO_2_ in RPMI-1640 (Nacalai Tesque, Kyoto, Japan) supplemented with 10% Newborn Calf Serum (Thermo Fisher Scientific, Waltham, MA, USA).

### Mice

Male 5- to 7-week-old C3H/HeN mice were obtained from Charles River Japan (Kanagawa, Japan) and CLEA Japan (Tokyo, Japan). All animals were allowed to acclimatize and recover from shipping-related stress for 1 week prior to the study. The health of the mice was monitored by daily observation. The animals were allowed free access to chlorinated water and irradiated food, and were kept in a controlled light–dark cycle (12 h–12 h). Animal procedures were approved by the Institutional Animal Care and Use Committee at Chugai Pharmaceutical Co., Ltd., and conformed to the *Guide for the Care and Use of Laboratory Animals* published by the Institute of Laboratory Animal Resources (ILAR).

### Tumor model

FM3A tumor cells (1.0 × 10^6^) in 100 μL of culture medium were subcutaneously inoculated into the left flank of C3H/HeN mice. The administration of anticancer agents was started when the tumor volumes reached approximately 30 to 220 mm^3^ (Day 1). Anti-mouse PD-L1 mAb (10F.9G2; BioLegend, San Diego, CA, USA) or Rat IgG (MP Biomedicals, Santa Ana, CA, USA) was administered intraperitoneally to the mice at a dose of 10 mg/kg three times a week. Anti-mouse FR4 mAb (TH6; BioLegend) or Rat IgG (MP Biomedicals) was administered intraperitoneally at a dose of 10 µg/head twice a week. Irinotecan (Daiichi Sankyo, Tokyo, Japan) or saline was administered intraperitoneally at a dose of 250 mg/kg on Day1.

Tumor volume (*V*) was estimated from the equation *V* = *L* × *W*^2^ × 0.5 (*L* = length; *W* = width). The joint action of the irinotecan plus PD-L1 mAb combination was compared to the actions of the individual agents by examining the mean response of each group, where the response variable was the relative tumor volume (tumor volume divided by its initial volume) at the end of the study (Day 19).

The joint action is additive if the relative effect of the drug combination equals the multiplied relative effects of the two single drugs; that is, if

(mean response of PD-L1 mAb plus irinotecan group)/(mean response of control group) = [(mean response of irinotecan group)/(mean response of control group)] × [(mean response of PD-L1 mAb group)/(mean response of control group)]

The joint action is supra-additive if

(mean response of PD-L1 mAb plus irinotecan group)/(mean response of control group) < [(mean response of irinotecan group)/(mean response of control group)] × [(mean response of PD-L1 mAb group)/(mean response of control group)].

### Flow cytometric analysis

For analysis of tumor-infiltrating lymphocytes, tumor tissue was excised from control-treated mice and anticancer agent-treated mice, and single cell suspensions were obtained by mincing tumors and homogenizing them by disruption and digestion with a gentleMACS Dissociator and a Tumor Dissociation Kit for mice (Miltenyi Biotec, Bergisch Gladbach, Germany). For analysis of lymph nodes, lymphocytes from axillary and brachial lymph nodes on the right side of tumor-bearing mice were harvested and mixed. For analysis of peripheral blood, blood was pretreated with VersaLyse lysing solution for red blood cell lysis (Beckman Coulter, Brea, CA, USA). Single cell suspensions were incubated with anti-Fcγ receptor (Tombo Biosciences, San Diego, CA, USA) and the fixable viability dye FVD506 (eBioscience, San Diego, CA, USA) at 4° C for 10 minutes, and stained with the following monoclonal antibodies: mouse CD45 (30-F11), CD4 (RM4-5), CD8α (53–6.7), CD69 (H1.2F3), CD11b (M1/70), Gr-1 (RB6-8C5), CD11c (HL3), H-2D^k^ (15-5-5), PD-L1 (MIH5), F4/80 (T45-2342), Foxp3 (FJK-16s), and Ki-67 (B56) from BioLegend or BD Biosciences (Franklin Lakes, NJ, USA). The appropriate conjugated isotype-matched immunoglobulin Gs (IgGs) were used as the control for each. Intracellular cytokine staining was performed with the use of a Foxp3/Transcription Factor Staining Buffer Set (eBioscience). Cells were analyzed using an LSRFortessa X-20 cell analyzer (BD Biosciences) and FlowJo 10 software (Tree Star, San Carlos, CA, USA).

For general hematologic analysis of the blood, neutrophils, lymphocytes, monocytes, and eosinophils were measured with an automated hematology analyzer (XT-2000iV; Sysmex, Hyogo, Japan).

### Tumor-specific IFNγ release assay

Tumor-draining lymph nodes were assessed on Day 19 for specific antitumor response by analyzing IFNγ release. Lymphocytes from axillary, brachial, and inguinal lymph nodes on the right side of tumor-bearing mice were harvested and mixed. These lymphocytes were co-cultured with irradiated tumor cells in a 10:1 ratio (lymphocyte/tumor cells) at 37° C for 3 days. FM3A cells were the target cells, and that MBT-2 cells were used as a negative control. IFNγ in the culture supernatant was examined by ELISA (R&D Systems, Minneapolis, MN, USA).

### Immunohistochemistry

We evaluated the localization of CD8^+^ T cells in tumor tissue by immunohistochemical staining of CD8α (rat anti-mouse CD8α mAb, KT15 from GeneTex (Irvine, CA, USA)). Tumor samples were collected at the end of the study (Day 19).

### *In vitro* PD-L1 and H-2D^k^ expression assay

Cells were seeded at a density of 5 × 10^5^ cells/well in 24-well plates. The cells were then treated with SN-38, the active metabolite of irinotecan, for 24 hours. Cells were collected and stained with the following monoclonal antibodies: mouse PD-L1 (MIH5) and H-2D^k^ (15-5-5). The appropriate conjugated isotype-matched IgGs were used as control for each. Cells were analyzed using an LSRFortessa X-20 cell analyzer and FlowJo 10 software.

### Statistical analysis

To evaluate statistical significance, data was analyzed with Wilcoxon test. For two groups, *p* < 0.05 was considered to indicate a significant difference. The method of Holm [28] was used to adjust the *P* values in multiple testing using JMP version 10 software (SAS Institute, Cary, NC, USA). For example, when reporting *P* values for K distinct tests, the Holm method is to compare the rth smallest *P* value (for r = 1, . . . , K) among the *P* values with 0.05/(K − r + 1), and the test result is considered statistically significant after adjustment for the multiple tests if the rth smallest *P* value is less than 0.05/(K − r + 1). However, if the rth smallest *P* value is the first that exceeds 0.05/(K − r + 1), then the test results associated with the (K − r + 1) largest *P* values are considered statistically nonsignificant according to the Holm method.

## Abbreviations

PD-L1: programmed death ligand 1
PD-1: programmed cell death 1
FR4: folate receptor 4
mAb: monoclonal antibody
Tregs: regulatory T cells
MDSCs: myeloid-derived suppressor cells
